# Lack of direct evidence for natural selection at the candidate thrifty gene locus, *PPARGC1A*

**DOI:** 10.1186/s12881-016-0341-z

**Published:** 2016-11-15

**Authors:** Murray Cadzow, Tony R. Merriman, James Boocock, Nicola Dalbeth, Lisa K. Stamp, Michael A. Black, Peter M. Visscher, Phillip L. Wilcox

**Affiliations:** 1 Department of Biochemistry, University of Otago, Dunedin, New Zealand; 2 Virtual Institute of Statistical Genetics (www.visg.co.nz), Dunedin, New Zealand; 3 formerly Scion (New Zealand Forest Research Institute Ltd), 49 Sala Street, Rotorua, New Zealand; 4 Department of Medicine, University of Auckland, Auckland, New Zealand; 5 Department of Medicine, University of Otago, Christchurch, New Zealand; 6 The Queensland Brain Institute, University of Queensland, Brisbane, Australia; 7 University of Queensland Diamantina Institute, University of Queensland, Translational Research Institute (TRI), Brisbane, Australia; 8 Department of Mathematics and Statistics, University of Otago, Science III Building, 730 Cumberland St, Dunedin, 9016 New Zealand

**Keywords:** Natural selection, Selective sweeps, Thrifty gene, Gout, Type 2 diabetes, BMI

## Abstract

**Background:**

The gene *PPARGC1A*, in particular the Gly482Ser variant (rs8192678), had been proposed to be subject to natural selection, particularly in recent progenitors of extant Polynesian populations. Reasons include high levels of population differentiation and increased frequencies of the derived type 2 diabetes (T2D) risk 482Ser allele, and association with body mass index (BMI) in a small Tongan population. However, no direct statistical tests for selection have been applied.

**Methods:**

Using a range of Polynesian populations (Tongan, Māori, Samoan) we re-examined evidence for association between Gly482Ser with T2D and BMI as well as gout. Using also Asian, European, and African 1000 Genome Project samples a range of statistical tests for selection (*F*_ST_, integrated haplotype score (iHS), cross population extended haplotype homozygosity (XP-EHH), Tajima’s *D* and Fay and Wu’s *H*) were conducted on the *PPARGC1A* locus.

**Results:**

No statistically significant evidence for association between Gly482Ser and any of BMI, T2D or gout was found. Population differentiation (*F*_ST_) was smallest between Asian and Pacific populations (New Zealand Māori ≤ 0.35, Samoan ≤ 0.20). When compared to European (New Zealand Māori ≤ 0.40, Samoan ≤ 0.25) or African populations (New Zealand Māori ≤ 0.80, Samoan ≤ 0.66) this differentiation was larger. We did not find any strong evidence for departure from neutral evolution at this locus when applying any of the other statistical tests for selection. However, using the same analytical methods, we found evidence for selection in specific populations at previously identified loci, indicating that lack of selection was the most likely explanation for the lack of evidence of selection in *PPARGC1A*.

**Conclusion:**

We conclude that there is no compelling evidence for selection at this locus, and that this gene should not be considered a candidate thrifty gene locus in Pacific populations. High levels of population differentiation at this locus and the reported absence of the derived 482Ser allele in some Melanesian populations, can alternatively be explained by multiple out-of-Africa migrations by ancestral progenitors, and subsequent genetic drift during colonisation of Polynesia. Intermediate 482Ser allele frequencies in extant Western Polynesian populations could therefore be due to recent admixture with Melanesian progenitors.

**Electronic supplementary material:**

The online version of this article (doi:10.1186/s12881-016-0341-z) contains supplementary material, which is available to authorized users.

## Background

The thrifty gene theory is based on the hypothesis that alleles causing increased weight, insulin resistance and type 2 diabetes (T2D) in contemporary populations were an adaptation to fluctuating food availability [1], for example from extreme environmental events and extended sea travel, in ancestral populations. Any genuine thrifty gene variant would have been selectively advantageous and therefore subject to selection during human evolution. Thrifty genetic variants arising early in hominid evolution, for example the uricase knockout [2], would be expected to be monomorphic and shared by all human populations. However, candidate thrifty variants arising more recently in human evolution and polymorphic in contemporary populations should exhibit signatures of selection in the immediate genomic vicinity.

The *PPARGC1A* transcriptional regulator has a central role in insulin signalling and mitochondrial regulation [3]. The functional Gly482Ser (*rs8192678*) variant (reviewed in [4]) in the *PPARGC1A* gene is associated with body mass index (BMI) in a Tongan (Polynesian) population [4]. This led to the hypothesis that the Gly482Ser variant represents a candidate ‘thrifty variant’ in Pacific populations. This was based largely on the observation that the postulated thrifty (derived) 482Ser allele exhibited the highest prevalence world-wide in Polynesian populations, who also have high levels of T2D and BMI. In contrast, low T2D prevalence levels have been recorded in Papuan populations lacking the 482Ser allele [5]. This 482Ser allele observation also supported by a previous report of extreme *F*_ST_ values (a measure of inter-population difference at specific genetic variants) at the *PPARGC1A* locus [5]. To date however, this hypothesis has not been formally tested in a range of Polynesian populations using more direct assessments of natural selection.

The Māori and non-indigenous Polynesian populations of Aotearoa New Zealand (NZ) have a high prevalence of obesity, T2D and related metabolic conditions such as gout [6–8]. The reasons for this appear to be complex and are the result of a combination of poor environmental dietary exposure (contributed to by socioeconomic status) and inherited genetic variants [9]. However, extremely little is known about the etiology of obesity and T2D in NZ Māori and Pacific. If the *PPARGC1A* gene were associated with weight and subject to selection in Pacific populations, then this would be important knowledge on the aetiology of these conditions in contemporary populations. Furthermore, evidence for selection during Polynesian population history would aid in the destigmatisation of obesity. Here, we tested for association between Gly482Ser rs8192678, BMI, T2D and gout in several Polynesian populations. We also examined evidence for recent positive selection at the derived 482Ser allele and in the region of *PPARGC1A*, by applying a recently developed analytical pipeline [10] to genomic data from the various Polynesian and 1000 Genomes Project world-wide populations.

## Methods

### Populations

New Zealand (NZ) Māori, Cook Island Māori, Samoan, Tongan and NZ Caucasian sample sets were examined (Table 1). Samples were collected as part of the “Genetics of Gout in Aotearoa” case–control study [11] and ethical approval was obtained from the New Zealand Multi-region Ethics committee (MEC/105/10/130). Each participant provided informed written consent. Patients with gout satisfied, by clinical examination, the American Rheumatism Association preliminary criteria for acute gout [12]. BMI and self-reported T2D status was recorded at time of recruitment. Subjects were classified into ancestry groups based on self-reported ancestry of grand-parents. Table 1 provides the number of samples in each population, and the methods used for genotyping and data analyses. Tables 2 and 3 describe clinical data and ancestry information for the NZ sample sets.

**Table 1:** Populations, genotyping platforms and analyses used to evaluate evidence of association and selection in *PPARGC1A*

		Phenotype		
Population	Genotyping Platform	Gout (*n*)	Control (*n*)	Analysis
NZ Caucasian	TaqMan SNP Assay	647	360	Single Marker GLM + F_ST_ on rs8192678
NZ Māori	TaqMan SNP Assay	137	176	Single Marker GLM + F_ST_ on rs8192678
CI Māori	TaqMan SNP Assay	71	64	Single Marker GLM + F_ST_ on rs8192678
Samoan	TaqMan SNP Assay	144	76	Single Marker GLM + F_ST_ on rs8192678
Tongan	TaqMan SNP Assay	73	41	Single Marker GLM + F_ST_ on rs8192678
Māori	AXIOM SNP chip	35	36	F_ST_, Fay and Wu’s H, Tajima’s D, iHS and XP-EHH
Samoan	OMNIexpress chip	48	48	F_ST_, Fay and Wu’s H, Tajima’s D, iHS and XP-EHH
		Unknown Phenotype		
1KGP CEU	WGS	99		F_ST_, Fay and Wu’s H, Tajima’s D, iHS and XP-EHH
1KGP CHB	WGS	103		F_ST_, Fay and Wu’s H, Tajima’s D, iHS and XP-EHH
1KGP CHS	WGS	108		F_ST_, Fay and Wu’s H, Tajima’s D, iHS and XP-EHH
1KGP GBR	WGS	92		F_ST_, Fay and Wu’s H, Tajima’s D, iHS and XP-EHH
1KGP YRI	WGS	109		F_ST_, Fay and Wu’s H, Tajima’s D, iHS and XP-EHH

All individuals in the Māori AXIOM and Samoan OMNIexpress datasets were also genotyped by the TaqMan® assay, and included in their respective population groups. NZ = New Zealand. CI = Cook Islands. WGS = whole genome sequencing. 1KGP = 1000 genomes project (http://www.1000genomes.org/home). Populations used were Utah, USA residents with Northern and Western European ancestry (CEU, Han Chinese in Beijing, China (CHB), Southern Han Chinese (CHS), China, British in England and Scotland (GBR) and Yoruba in Ibadan, Nigeria YRI. For a complete description of the 1000 Genomes project samples see http://www.1000genomes.org/data#DataAvailable. No phenotypic data were available for these populations

**Table 2:** Clinical and genetic information for sample sets used for association analysis with BMI, T2D and gout

	obs missing (*n*)	NZ Caucasian	obs missing (*n*)	NZ Māori	obs missing (*n*)	CI Māori	obs missing (*n*)	Samoan	obs missing (*n*)	Tongan
Individuals	-	1007	-	322	-	136	-	223	-	114
Gout (%)	-	64.3	9	42.5	1	52.2	3	64.6	-	64.0
Diabetes (%)	10	11.5	11	42.8	4	26.5	6	14.3	3	18.4
Mean age (range)	-	59.7 (18–94)	1	50.9 (17–82)	-	52.4 (18–88)	-	44.3 (17–71)	-	42.8 (18–79)
Sex (% male)	2	76.1	1	56.8	-	61.8	2	82.1	-	86.8
Mean BMI Gly/Gly (range)	6	29.1 (17.8–61.6)	1	32.3 (23.0–42.2)	2	35.9 (33.1–40.7)	-	37.2 (26.0–62.5)	-	34.5 (26.3–44.2)
Mean BMI Gly/Ser (range)	9	29.3 (18.8–60.7)	3	33.0 (21.3–61.7)	-	34.5 (21.4–66.7)	3	37.7 (22.2–93.4)	-	36.5 (26.6–48.5)
Mean BMI Ser/Ser (range)	-	29.7 (19.3–62.9)	3	34.2 (18.4–65.8)	1	36.5 (23.0–80.0)	1	36.7 (20.5–70.3)	1	36.8 (25.8–70.6)
Allele frequency rs8192678 Ser allele (Gout; Control)	2	0.346 (0.332; 0.371)	2	0.801 (0.803; 0.798)	-	0.805 (0.782; 0.836)	-	0.688 (0.698; 0.671)	-	0.636 (0.651; 0.61)
Allele frequency rs8192678 Ser allele (Diabetes; Control)	2	0.346 (0.366; 0.345)	2	0.801 (0.788; 0.807)	-	0.805 (0.847; 0.786)	-	0.688 (0.703; 0.689)	-	0.636 (0.667; 0.622)

*BMI* body mass index, *Gly* glycine, *Ser* serine

**Table 3:** Clinical and ancestral information for sample sets used in tests for selection analysis

	obs missing (n)	Māori AXIOM	obs missing (n)	Samoan OMNIexpress
Number	-	71	-	96
Sex (% male)	-	64.7	-	74
Gout cases (%)	-	49.3	-	50.0
Mean BMI (range)	1	34.5 (22.1 to 56.1)	-	36.1 (20.5 to 93.4)
Diabetes (%)	3	21.1	-	12.5
Mean grandparent ancestry (range)	-	0.916 (0.625 to 1.00)	-	0.997 (0.875 to 1.00)

Mean grandparent ancestry is the proportion of grandparents self-reported as belonging to the ethnicity group

*BMI* body mass index

Five additional populations were obtained from the 1000 Genomes Project phase 3 data release (see [13]): two Asian populations: Han Chinese in Beijing, China (CHB), Southern Han Chinese, China (CHS); two of European ancestry: British in England and Scotland (GBR) and Utah residents with Northern and Western European ancestry (CEU); and one of African ancestry: Yoruba in Ibadan, Nigeria (YRI). The CHB and CHS populations are intermediate with regard to Gly482Ser frequencies between the African (YRI) and European (GBR, CEU) on the one hand and the Polynesian populations on the other [14].

### Genotypic data

Two genotypic data sets were used for this study: (a) a SNP-specific assay corresponding to a Glycine (C allele) -Serine (T allele) substitution at amino acid position 482 in the *PPARGC1A* gene (dbSNP ID rs8192678 residing on chromosome 4 (GRCh37 chr4:23815662) and genotyped on a subset of the above populations (see Table 1); and (b) chromosome 4-wide genotypic data. For (a) SNP rs8192678 was genotyped with TaqMan® assay ID C___1643192_20 (Life Technologies, Carlsbad, CA) using a LightCycler 480 Real-Time PCR System (Roche Applied Science, Indianapolis, IN). The MINI MELT program was run from within the LightCycler software, and genotypes were assigned based on clustering within the software. Clusters were visually checked with misassigned genotypes corrected where appropriate or designated as unknown. 9% of samples were re-genotyped and cross checked as a quality control measure. All re-genotyped samples had complete agreement with the original genotypes. For (b) genotypic data for whole chromosome analyses were obtained either directly from SNP microarrays or whole genome sequencing. For the SNP microarrays, 71 individuals who self-reported four Māori grandparents were genotyped using an Affymetrix AXIOM genome-wide ASI array (Table 1). Similarly, 96 individuals self-reporting four Samoan grandparents were genotyped with an Illumina Human OmniExpress bead chip. The individuals genotyped by SNP microarray were a subset from those genotyped with the Taqman® assay. Whole genome sequence (WGS) from 1000 Genomes Project phase 3 data release (via [13]) was used.

### Association analyses

To determine associations between BMI and rs8192678 genotype, single marker linear regressions were undertaken using the R statistical software environment [15]. Hardy-Weinberg equilibrium exact tests were calculated using the R package HardyWeinberg [16]. For each population, three modes of gene action were tested: additive, dominant (both C and derived T allele), and over-dominant. Age, sex, gout affection status, T2D, and Structure-estimated ancestry proportions (calculated as described in [11]) were included as covariates for BMI. For T2D and gout affection status, logistic regression was performed with the same covariates. A meta-analysis of the Polynesian populations was undertaken by combining the NZ Māori, Cook Island Māori, Tongan and Samoan populations and repeating the marker trait regressions as described above with the added covariate of population.

### Selection analyses

Pairwise *F*_ST_ [17] was estimated for all populations for rs8192678. For populations where genome-wide genotypic data were available (i.e., the 1000 Genomes Project and Axiom/Omni-genotyped populations, Table 1), the following statistics were calculated: *F*_ST_ between sample sets, Tajima’s *D*, Fay and Wu’s *H*, and integrated haplotype score (iHS, [18]) for individual populations, and cross population haplotype homozygosity (XP-EHH, [19]) to estimate selection between populations. To calculate these statistics we used a customized analytical pipeline [10]. For these analyses we assumed that the 482Ser allele was the derived allele, based on low frequencies of this allele in African populations [5]. *F*_ST_ was calculated for the entire chromosome using the Weir and Cockerham method with negative values manually set to zero [17]. Quantiles of 2.5 and 97.5% were used to find the most extreme 5% of values. Tajima’s *D* [20] and Fay and Wu’s *H* [21] were calculated for the whole chromosome using bins of 1, 5 and 30 kbp. The Tajima’s *D* for the entire chromosome with thresholds of the 2.5 and 97.5% quantiles used to establish the most extreme 5% of values.

The software package *selscan* [22] was used to calculate iHS and XP-EHH with values for both being normalised in frequency bins genome-wide. Cross population extended haplotype homozygosity was used to detect selection of alleles at or near fixation, and was calculated between populations as described in [19]. An iHS or XP-EHH absolute value of greater than 3.29 was used as a threshold to estimate the most extreme 1% of values from the score distribution. The iHS normalisation conformed to a standard Gaussian distribution for all populations. For XP-EHH, all pairwise comparisons were performed between the groups of populations that had been genotyped with genome-wide SNP arrays or via WGS.

Fourteen combinations of populations and genes (corresponding to six genes) previously reported by Voight et al. [18] as showing evidence of selection were analysed as positive controls to provide insight into the overall power of detecting signatures of selection using the methods described above (Additional file 1: Table S1).

## Results

### Association analyses

Tests for association between BMI and rs8192678 genotype did not reveal any statistically significant association for any of the gene action models (Table 4). Similarly, no relationship was observed between rs8192678 genotype and either gout affection or T2D status, for any of the modes of genetic action (Table 4). There were also no statistically significant associations observed for the meta-analysis of the Polynesian populations between BMI, or gout affection, or T2D status and rs8192678 under any of the modes of genetic action.

**Table 4:** Results from a single marker generalised linear model for rs8192678 and BMI, T2D and gout

	Allele Freq (T)	HWE	Additive	*P* value	Dominant C (Gly)	*P* value	Dominant T (Ser)	*P* value	Heterozygous Advantage	*P* value
Linear regression between BMI and rs8192678 under the 4 genetic models. Beta (95% CI)										
NZ Caucasian	0.346	0.728	0.319 (−0.198, 0.835)	0.227	−0.555 (−1.618, 0.507)	0.305	0.350 (−0.358, 1.058)	0.332	0.102 (−0.601, 0.806)	0.775
NZ Māori	0.806	0.721	0.629 (−0.848, 2.106)	0.403	−0.837 (−2.588, 0.913)	0.347	0.263 (−3.885, 4.412)	0.901	−0.831 (−2.625, 0.964)	0.363
CI Māori	0.805	1.000	0.612 (−2.342, 3.566)	0.682	−0.627 (−3.940, 2.686)	0.709	1.319 (−8.733, 11.371)	0.795	−0.504 (−3.886, 2.879)	0.769
Samoan	0.688	0.043	−0.610 (−2.605, 1.386)	0.548	0.705 (−1.757, 3.166)	0.573	−0.873 (−5.749, 4.002)	0.724	0.487 (−1.989, 2.964)	0.698
Tongan	0.636	0.310	0.755 (−0.958, 2.468)	0.384	−0.895 (−3.382, 1.592)	0.477	1.336 (−2.118, 4.791)	0.445	−0.218 (−2.799, 2.363)	0.867
Polynesian	0.747	0.925	0.345 (−0.611, 1.300)	0.479	−0.408 (−1.587, 0.770)	0.496	0.470 (−1.904, 2.843)	0.698	−0.301 (−1.496, 0.895)	0.621
Logistic regression between gout affection and rs8192678. OR (95% CI)										
NZ Caucasian	0.346	0.728	0.846 (0.683, 1.047)	0.125	1.290 (0.828, 1.996)	0.256	0.819 (0.612, 1.095)	0.180	0.916 (0.686, 1.221)	0.548
NZ Māori	0.806	0.721	1.238 (0.737, 2.111)	0.425	0.896 (0.488, 1.635)	0.721	3.644 (0.697, 28.949)	0.159	1.071 (0.578, 1.977)	0.827
CI Māori	0.805	1.000	0.785 (0.361, 1.654)	0.529	1.126 (0.488, 2.611)	0.780	-	-	0.936 (0.397, 2.202)	0.879
Samoan	0.688	0.043	1.110 (0.638, 1.933)	0.711	1.097 (0.543, 2.220)	0.796	2.328 (0.658, 8.556)	0.189	1.437 (0.707, 2.975)	0.320
Tongan	0.636	0.310	1.073 (0.562, 2.039)	0.829	1.248 (0.477, 3.310)	0.651	1.911 (0.543, 6.737)	0.308	1.991 (0.713, 5.971)	0.199
Polynesian	0.747	0.925	1.149 (0.862, 1.533)	0.345	0.970 (0.678, 1.385)	0.865	2.024 (1.005, 4.126)	0.049	1.170 (0.814, 1.685)	0.396
Logistic regression between T2D and rs8192678. OR (95% CI)										
NZ Caucasian	0.346	0.728	1.046 (0.767, 1.419)	0.774	0.953 (0.523, 1.835)	0.880	1.065 (0.700, 1.630)	0.771	1.041 (0.686, 1.576)	0.850
NZ Māori	0.806	0.721	0.768 (0.463, 1.294)	0.311	1.362 (0.737, 2.495)	0.319	0.706 (0.181, 3.535)	0.636	1.299 (0.692, 2.407)	0.409
CI Māori	0.805	1.000	2.280 (0.901, 6.410)	0.096	0.348 (0.111, 0.977)	0.054	0.647 (0.037, 20.494)	0.777	0.314 (0.095, 0.910)	0.042
Samoan	0.688	0.043	1.307 (0.630, 2.814)	0.480	0.788 (0.329, 1.886)	0.590	2.043 (0.316, 40.719)	0.527	0.904 (0.379, 2.142)	0.818
Tongan	0.636	0.310	1.063 (0.477, 2.443)	0.882	0.961 (0.328, 2.888)	0.942	1.198 (0.233, 9.316)	0.841	1.030 (0.342, 3.045)	0.957
Polynesian	0.747	0.925	1.738 (0.768, 4.219)	0.200	0.911 (0.610, 1.335)	0.646	1.104 (0.476, 2.901)	0.827	0.928 (0.617, 1.386)	0.716

The marker was assessed under an additive model, dominance models and heterozygous advantage model. Models are adjusted for gout and diabetes affection, sex, age and non –Caucasian ancestry. Estimate (95% CI). NZ = New Zealand. CI = Cook Islands. Polynesian is a combination of NZ Māori, CI Māori, Samoan, and Tongan population groups. HWE = *P*-value for departure from Hardy Weinberg expectations using exact test, Logistic regression for CI Māori under the dominant T model could not be performed

### Selection analyses

#### Intra-population tests for selection – Tajima’s D, Fay and Wu’s H and iHS

Estimates of Tajima’s *D* and Fay and Wu’s *H* for windows surrounding the Gly482Ser position, were within +/− 2.5% limits for all populations and window lengths (Tables 5 and 6). Only the two Chinese populations (CHB and CHS) had strong positive values for Tajima’s *D* in the 1kbp region surrounding, suggesting possible balancing selection, however estimates decayed to values close to those of the chromosome average for the 30kbp window size (Table 5), and were well within +/− 2.5% thresholds. For Tajima’s *D* in 1 kb windows that overlapped the *PPARGC1A*-encoding region, all populations had windows that exceeded both +2.5 and −2.5% thresholds (Additional file 2: Figure S1c), but for the 5 kb windows only the Māori population had windows exceeding the lower threshold (Additional file 2: Figure S1b), indicating the possibility of a selective sweep. However, no population had any window in the *PPARGC1A* genic region exceeding either threshold for the 30kbp region for Tajima’s *D* (Additional file 2: Figure S1a). Moreover, there did not appear to be a clear pattern within any population that was consistent with a selective sweep: estimates for the different window sizes were typically dispersed around the chromosome mean for each population rather than showing a pattern where values consistently exceeded the chromosome thresholds. Similarly, for Fay and Wu’s *H*, no windows overlapping the *PPARGC1A* region that exceeded +/− 2.5% thresholds were observed with windows of 30 kbp (Additional file 3: Figure S2a). Only the Samoan population had a window that exceeded the threshold in the *PPARGC1A* genic region for the 5 kb windows (Additional file 3: Figure S2b). For the 1 kb windows, both the Māori and Samoan populations had regions that exceeded lower thresholds, indicating the possibility of an excess of high frequency derived alleles (Additional file 3: Figure S2c). However, for the region containing rs8192678, no window exceeded the +/− 2.5% thresholds in any population, irrespective of window size (Table 6). Neither of these site frequency spectra-based tests therefore revealed evidence of selection in the regions containing the Gly482Ser substitution.

**Table 5:** Tajima’s *D* at *PPARGC1A* calculated with window sizes of 1 kbp, 5 kbp, and 30 kbp

	Tajima’s D	2.5% Quantile	Chromosome Mean	97.5% Quantile
Tajima’s D value for 1 kb window containing rs8192678				
CEU	0.810	−1.127	0.772	3.053
CHB	2.400	−0.835	1.068	3.199
CHS	2.566	−0.835	1.104	3.218
GBR	0.924	−1.091	0.817	3.068
YRI	−0.445	−1.193	0.233	2.606
Māori	−0.080	−1.083	0.886	2.996
Samoan	0.622	−1.119	0.943	3.103
Tajima’s D value for 5 kb window containing rs8192678				
CEU	1.010	−1.358	1.089	3.805
CHB	1.729	−1.068	1.538	4.168
CHS	1.923	−1.054	1.599	4.204
GBR	1.846	−1.299	1.153	3.865
YRI	−0.150	−1.486	0.310	2.892
Māori	−1.040	−1.295	1.275	3.905
Samoan	−0.235	−1.383	1.363	4.074
Tajima’s D value for 30 kb window containing rs8192678				
CEU	1.480	−1.711	1.489	4.052
CHB	2.585	−1.265	2.102	4.730
CHS	2.267	−1.175	2.201	4.788
GBR	1.963	−1.571	1.567	4.185
YRI	0.029	−1.693	0.432	2.693
Māori	−1.098	−1.463	1.732	4.416
Samoan	−0.299	−1.698	1.857	4.616

Mean, 2.5% quantile and 97.5% quantile were calculated for chromosome 4 by population

**Table 6:** Fay’s and Wu’s H at *PPARGC1A* calculated using window sizes of 1 kbp, 5 kbp, and 30 kbp

	Fay and Wu’s H	2.5% Quantile	Chromosome Mean	97.5% Quantile
Fay and Wu’s H 1 kb window containing rs8192678				
CEU	−0.094	−2.549	−0.168	0.710
CHB	−0.106	−2.504	−0.174	0.691
CHS	−0.007	−2.511	−0.173	0.693
GBR	−0.227	−2.531	−0.162	0.713
YRI	−1.132	−2.345	−0.055	0.888
Māori	−0.845	−2.754	−0.217	0.648
Samoan	−0.345	−2.884	−0.223	0.667
Fay and Wu’s H 5 kb window containing rs8192678				
CEU	−0.689	−8.467	−0.839	2.018
CHB	−0.656	−8.237	−0.870	1.953
CHS	−0.754	−8.133	−0.865	1.952
GBR	−0.286	−8.309	−0.811	2.025
YRI	0.091	−7.300	−0.276	2.719
Māori	−3.996	−9.132	−1.085	1.778
Samoan	−2.342	−9.468	−1.117	1.859
Fay and Wu’s H 30 kb window containing rs8192678				
CEU	2.955	−36.264	−5.032	7.377
CHB	3.675	−35.444	−5.221	7.381
CHS	2.617	−35.894	−5.187	7.302
GBR	3.433	−35.387	−4.867	7.472
YRI	−1.989	−29.990	−1.657	10.606
Māori	−14.842	−3.826	−6.509	6.379
Samoan	−5.607	−41.697	−6.702	6.870

Mean, 2.5% quantile and 97.5% quantile were calculated for chromosome 4 by population

**Fig. 1:**
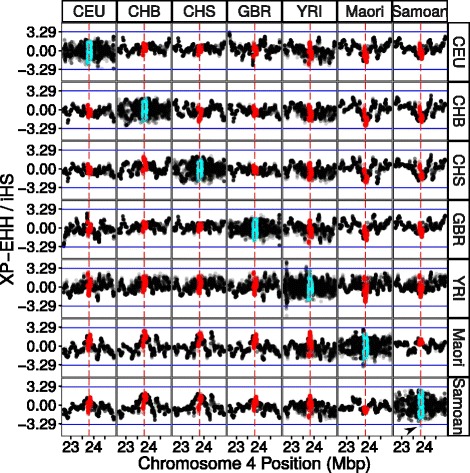
iHS/XP-EHH by Population. A +/− 1 Mb window centred on *PPARGC1A*.Threshold of |3.29| for both XP-EHH and iHS is marked with *blue lines*. Location of *PPARGC1A* is marked in *red* for XP-EHH and *light blue* for iHS. Rs8192678 is indicated by the *red dashed line*. iHS (within population) is shown on the diagonal. The arrowhead indicates the iHS value for rs8192678 in Samoans that exceeds the 1% threshold

We also calculated integrated haplotype homozygosity score (iHS) statistics for rs8192678 for each population using a genetic map. The iHS is a statistic to detect evidence of recent positive selection at a locus and is based on the differential levels of linkage disequilibrium surrounding a positively selected allele compared to the other allele at the same position. Only the Samoan population had |iHS| > 3.29 when computed using a genetic map (corresponding to the most extreme 1% of |iHS| values) (Table 7, Fig. 1). Plots of iHS +/− 1 Mbp of the *PPARGC1A*-encoding region showed relatively few locations within this region that exceeded the 3.29 threshold in any of the populations investigated (Fig. 1).

**Table 7:** iHS and XP-EHH statistics for *rs8192678*

	CEU	CHB	CHS	GBR	YRI	Māori	Samoan
CEU	−2.510^a^	0.548	0.163	0.321	1.319	−1.579	−0.771
CHB	−0.548	−1.912^a^	−1.578	−0.392	0.492	−2.695	−1.705
CHS	−0.163	1.578	−1.775^a^	−0.008	1.090	−2.226	−1.205
GBR	−0.321	0.392	0.008	−2.074^a^	0.795	−1.752	−0.983
YRI	−1.319	−0.492	−1.090	−0.795	-^a^	−2.565	−1.754
Māori	1.579	2.695	2.226	1.752	2.565	−3.101^a^	1.163
Samoan	0.771	1.705	1.205	0.983	1.754	−1.163	−3.560^a^

All values are given as XP-EHH with the exception of those denoted by ^a^indicating integrated haplotype homozygosity scores (iHS). Rs8192678 had a derived allele frequency < 0.05 in YRI and therefore iHS was not calculated

We also evaluated six genes that had previously been reported as exhibiting evidence of selection by Voight et al. (2006) in specific ancestries. Here, there were 14 combinations of genes and populations (Additional file 1: Table S1) since two ancestries – Asian and Caucasian – were each represented by two populations each in this study (CHS and CHB for Asians, and CEU and GBR for Caucasians – see Additional file 1: Table S1). Seven of the 14 gene-population combinations showed evidence of selection in the 1000 Genomes populations samples used for this study (where we define evidence of selection as >1 SNP exceeding the 1% threshold of iHS values, Additional file 1: Table S1). Overall, the analytical methods used in this study detected evidence of selection at half of the loci previously shown to be under selection [18] that were tested.

#### Inter-population tests for selection - *F*_ST_ and XP-EHH

Population differentiation at rs8192678 was investigated by calculating *F*_ST_ statistics for all pairwise combinations all populations that had been genotyped with the TaqMan® assay. Derived allele frequencies from the WGS and chip datasets were very similar to those found in Myles et al. [4] with Polynesian (NZ Māori 0.83, Samoan 0.72), Asian (CHB 0.37, CHS 0.45), European (CEU 0.37, GBR 0.33) and African (YRI 0.04). Results showed strong differentiation between NZ Caucasian and all of the Polynesian populations, with NZ Māori and Caucasian being the most differentiated (0.332, Table 8). Tongan and Samoan populations were less differentiated from the NZ Caucasian population (0.160 and 0.210 respectively). East – West Polynesia differentiation [23] was also apparent, with *F*_ST_ between NZ Māori and Samoan (0.033) and NZ Māori and Tongan (0.069) populations greater than NZ Māori and CI Māori (0.000). The Samoan and Tongan populations had relatively small differentiation between them (*F*_ST_ = 0.003). Population differentiation was also estimated for the Gly482Ser variants in the genome-wide data sets (Table 9). For the Gly482Ser substitution, Māori were more strongly differentiated than the Samoan population from all of the 1000 Genomes populations, with strongest differentiation occurring in the Yoruban population followed by the European and Chinese populations. A similar but less extreme trend was observed for the Samoan population: *F*_ST_ was highest for the Yoruban population, with decreasing levels of differentiation between European and Chinese populations respectively. Lower *F*_ST_ between Samoan and Caucasian populations compared to the Māori and Caucasian populations was observed in both the genome-wide genotyped populations and the TaqMan®-genotyped populations.

**Table 8:** Single marker *F*_ST_ for rs8192678

	NZ Caucasian	NZ Māori	CI Māori	Samoan
NZ Māori	0.334	-		
CI Māori	0.324	0.000	-	
Samoan	0.205	0.035	0.032	-
Tongan	0.154	0.073	0.066	0.003

F_ST_ on the same populations was not calculated and negative F_ST_ was set to zero

**Table 9:** Chromosome 4 *F*_ST_ for *rs8192678* in WGS and imputed chip data using 1 bp windows

	Māori				Samoan			
	Mean *F*_ST_ (window)	Mean *F*_ST_ (chromosome)	2.5% Quantile	97.5% Quantile	Mean *F*_ST_ (window)	Mean *F*_ST_ (chromosome)	2.5% Quantile	97.5% Quantile
CEU	0.373	0.095	0.000	0.436	0.222	0.099	0.000	0.433
CHB	0.348	0.062	0.000	0.320	0.199	0.044	0.000	0.216
CHS	0.259	0.061	0.000	0.306	0.122	0.042	0.000	0.201
GBR	0.404	0.095	0.000	0.435	0.249	0.100	0.000	0.434
YRI	0.802	0.137	0.000	0.591	0.655	0.134	0.000	0.572

We also calculated *F*_ST_ for 5Mbp windows surrounding the Gly482Ser location to determine the (genomic) extent of population differentiation (Table 10) over a wider window. Compared with the estimates at the *rs8192678* locus itself, 5Mbp window-based *F*_ST_ estimates were lower but still revealed the same trend of most differentiation between the Polynesian and Yoruban populations, with European populations the next most differentiated, and Chinese populations the least differentiated (Table 9). However, *F*_ST_ estimates between Māori and other genome-wide genotyped populations differed little from estimates between Samoan and other populations.

**Table 10:** Chromosome 4 *F*_ST_ for WGS and imputed chip data using 5 Mbp windows

	Māori				Samoan			
	Mean *F*_ST_ (window)	Mean *F*_ST_ (chromosome)	2.5% Quantile	97.5% Quantile	Mean *F*_ST_ (window)	Mean *F*_ST_ (chromosome)	2.5% Quantile	97.5% Quantile
CEU	0.094	0.094	0.068	0.120	0.084	0.098	0.070	0.133
CHB	0.069	0.061	0.038	0.088	0.050	0.044	0.027	0.067
CHS	0.067	0.060	0.035	0.090	0.047	0.042	0.025	0.067
GBR	0.088	0.094	0.067	0.124	0.082	0.099	0.070	0.133
YRI	0.145	0.138	0.106	0.178	0.142	0.136	0.098	0.188

Cross population extended haplotype homozygosity was calculated across chromosome 4 for all combinations of populations that had been resequenced or genotyped genome-wide. There was no combination of populations where XP-EHH estimates exceeded the |3.29| threshold in the *PPARGC1A* region (Table 7). Cross population extended haplotype homozygosity values were also plotted across the aforementioned region by pairwise population combination (Fig. 1).

## Discussion and conclusions

The primary aim of this study was to directly test the hypothesis that the *PPARGC1A* locus, in particular the Gly482Ser substitution, has been subject to natural selection in the progenitors of contemporary Polynesian populations. The appropriate basis for concluding a specific gene or region is subject to natural selection has been defined by Vitti et al. [24] as ‘*A combination of genomic and functional evidence constitutes the current standard for the field*’. In this study we combined association analyses between the Gly482Ser genotype and traits either directly (BMI) or indirectly (gout and T2D, which are correlated with BMI) to identify a potential functional role of Gly482Ser. To identify genomic evidence we used a recently-developed analytical pipeline to test selection by a combination of site frequency spectra based statistics, (Tajima’s *D*, Fay’s and Wu’s *H*) as well as haplotype-length based measures that examine selection within populations (iHS) or between populations (XP-EHH, [10]). We also estimated population differentiation (*F*_ST_), which has also been used as an indicator of selection [25], as well as departure from expected HW equilibrium. Of these various approaches, we were able to detect departures from the expected neutral selection model for *F*_ST_ only – no other statistics indicated consistent functional or genomic evidence for selection. However, reasons other than selection such as local co-ancestry can lead to outlier values for *F*_ST_ [26] therefore the *F*_ST_ results alone are not sufficient to conclude the presence of selection.

Lack of evidence of selection in other statistical tests may be a consequence of lack of power. In this regard, two factors are important in interpreting results from such analyses: (1) the nature of positive selection being investigated, and (2) power of each of the statistical tests to reject the null hypothesis of no departure from neutral model of selection. Regarding (1), two intra-specific selection scenarios were frequently examined: ‘hard sweeps’ within populations based on selective advantages arising from a *de novo* mutation with strong positive effects on fitness, and ‘soft sweeps’ based on extant variants underpinning heritable characteristics that are typically under polygenic control. Power (i.e., (2), above) to detect evidence of selection for the various methods used here differs depending upon these scenarios [27]. For the 482Ser allele investigated in this study, putative selection could be either via hard or soft sweeps. On the one hand, the previously reported absence of the derived 482Ser allele in some African and New Guinean populations [5] imply selection on *a de novo* mutation arising prior to ancestors of modern *Homo sapiens* migrating out Africa. On the other hand however, there is a relative dearth of evidence of hard sweeps in humans [28]. In addition, if *PPARGC1A* were indeed under selection, it would have been subject to concomitant selection in multiple Polynesian and other populations (e.g., [29]) and acting on extant variation at this locus. This suggests soft sweeps are a more likely scenario, especially as Gly482Ser is possibly one of very many small effect loci impacting the T2D and correlated conditions such as BMI [30, 31].

Of the methods used in this study to directly examine evidence of selection, none indicate selection has possibly occurred at this locus (Tables 4, 5, 6 and 7, and Fig. 1). The power of Tajima’s *D*, Fay’s and Wu’s *H*, and iHS to detect evidence of selection has recently been evaluated by Ferrer-Admetlla et al. [27] for both hard and soft sweeps. For Tajima’s *D* they reported variable power across a wide range of selection coefficients, window sizes, and final (derived) allele frequencies for hard sweeps. In hard sweeps where ending allele frequencies were similar to those observed in Māori populations (Table 4), moderate-high power was reported, but in soft sweeps power was relatively low (<0.2). Even if such tests are underpowered, the high number of populations, some of which are from the same ancestry (i.e., CEU and GBR being Caucasian, and CHB and CHS being Asian) would likely have detected selection should a hard sweep at this locus be occurring. Moreover, it is reasonable to assume that if the 482Ser allele were a genuine thrifty gene variant, selection would be occurring in all populations rather than being restricted to the Polynesian populations, as most ancestral populations would have experienced periodic limitations in food availability. For iHS, Ferrer-Admetlla et al. [27] reported power was generally high (>0.75) irrespective of whether hard or soft sweeps had occurred, and was similarly robust to different ending allele frequencies – which ranged from 0.5 to 0.9. The derived 482Ser allele frequency estimates in all of the Polynesian sample sets investigated in this study fall within this range.

To provide further insights into whether or not the lack of evidence of selection according to these statistics was due to inadequate sample sizes, we also analysed 14 population/gene combinations that had previously been identified as showing evidence of selection [18]. In our population samples, seven of these 14 combinations had >1 SNP exceeding the 1% iHS threshold, demonstrating that our methods could identify loci previously shown to be under selection (Additional file 1: Table S1). Moreover, in all of the seven cases, multiple SNPs exceeded this threshold, and for two genes, there was evidence of selection in both population samples representing the same ancestry (i.e., evidence of selection in both CEU and GBR populations for *LCT*, and in both CHB and CHS for *SLC445A5*, see Additional file 1: Table S1). This indicates that population samples of this size have approximately 50% power to detect an association in the 1000 Genomes populations – none of which were case–control studies - and that when selection is present, there should be multiple SNPs exceeding the threshold. Based on the above results, we therefore expect approximately (1–0.5^2^) ≈ 0.75 probability of at least one of the Samoan and Māori populations used in this study to show evidence of selection using the iHS test. However the (case–control) Samoan and Māori cohorts had (only) one and zero SNPs, respectively, exceeding the 1% iHS threshold. We also contend that, if anything, the use of a case–control design would be more likely to increase the frequency of haplotypes carrying the selected allele(s), and thus more likely to improve power should this locus be subject to selection. Therefore the lack of a statistically significant difference in 482Ser allele frequencies in the case control Māori cohort used in this study and the previously reported estimate by Myles et al. [5] from a cross-sectional study of a single Māori tribal group located in the East Coast of the North Island of NZ, is further evidence of the lack of evidence of natural selection at this locus.

A further consideration is the elevated derived 482Ser allele frequency in all non-African populations compared to the African populations [4, 5]. These show a progressive differentiation of populations at this locus. Thus, if *PPARGC1A* were a thrifty gene candidate that has been subjected to natural selection, selection would likely have occurred in all non-African ancestral populations due to factors such as climatic extremes, competition with other human groups, and/or the need to adapt to newly colonised environments - all of which are likely to contribute to periodic deprivations in food availability in progenitors of all populations studied.

The highest derived allele frequencies and strongest differentiation from extant African populations occurs in the Cook Islands and NZ Māori populations. Therefore if this locus is indeed a thrifty gene, recent migration histories would suggest that these populations would be likely to show evidence of association and/or selection as ancestors of these populations colonised East Polynesia less than 1500 years ago [32]. Thus we would expect repeated evidence of selection at this locus in most - if not all - of the populations examined. However, the reverse is the case: there is no strong evidence of positive selective at this locus according the iHS statistic, nor Tajima’s *D* nor Fay and Wu’s *H*. Similarly, for the NZ Māori population, evidence of possible differential selection indicated by Tajima’s *D* in the *PPARGC1A*-encoding region exceeding the lower 97.5% quantile, (Additional file 2: Figure S1a-c) was not reflected in either Fay and Wu’s H (Additional file 3: Figure S2a-c) or iHS (Fig. 1). Moreover, if selection on the derived 482Ser allele were occurring then this would be revealed by XP-EHH - for at least some population pairs. However, none of the XP-EHH exceeded threshold 1% values in any of the population pairs, nor were there any obvious differences in XP-EHH between pairs of populations that share ancestry (i.e., the CEU – GBR pair, and the CHB – CHS pair) compared to population pairs with different ancestries. Further, differences in derived 482Ser allele frequency within Polynesia – particularly those between Western Polynesian (i.e., Tongan and Samoan) and Eastern Polynesian (i.e., NZ and Cook Islands Māori) – are not explained by oceanic voyaging: the geographic distances between Tonga/Samoa and the Cook Islands is significantly less than the Cook Islands and NZ, yet the Cook Islands Māori 482Ser frequency differs from Samoa and Tonga and is virtually identical to NZ Māori. These differences are also reflected in the *F*_ST_ values (Table 8).

In addition, oral histories of Māori migrations make no reference to extensive loss of life on vaka/waka due to starvation during migration voyages [32], but do describe decisions by tribal groups, whose members were generally closely related (e.g., [33]), to migrate due to food competition and/or ongoing conflicts with other tribal groups - effectively increasing the possibility of genetic drift. We therefore conclude that the lack of evidence for selection in any of the statistical tests used in any of the populations examined in this study is likely an accurate biological reflection for the populations examined rather than a lack of statistical power to detect selective events at this locus with the methods used.

Existing 482Ser allele frequency distributions in the Pacific can be explained by a combination of (a) migration out of Africa by *Homo sapiens* progenitors and possibly Denisovians who either lacked the 482Ser allele or lost it via genetic drift (whose descendants include modern-day Melanesians such as Papuans), (b) later migration out of Africa by *Homo sapiens* progenitors of Polynesians subsequent to the mutation giving rise to the 482Ser allele, which drifted to increasingly higher frequencies in repeated migrations across the Pacific, and (c) followed by subsequent admixture between these Melanesian progenitors with ancestors of modern Polynesians [34]. The higher derived allele frequencies and genetic differentiation in Eastern Polynesians are likely the result of genetic drift in the ancestral population, possibly as a result of founder effects. This model is consistent with the absence of the 482Ser allele in Denisovian DNA sequences; hence any derived populations with Melanesian admixture – such as Tongan populations - would have lower frequencies than in Eastern Polynesian populations such as Cook Islands and NZ Māori. Genetic drift therefore could account for the progressive increase in allele frequency in modern non-African human populations, with founder effects arising from successive colonisation of islands within Polynesia resulting in the increase in 482Ser allele frequency.

Single marker regression analyses did not reveal any evidence of association between rs8192678 and BMI, gout affection status, or T2D in any of the populations investigated. Although each Polynesian population was small and likely to be underpowered for validating associations between small effect genes and conditions, no associations were found even when Polynesian populations were combined. We also found no evidence for sex-specific effects for any of the three traits in the combined Polynesian populations (data not shown). Our results contrast with those of Myles et al. [4] who reported associations for Tongan populations (*n* = 184, *P* = 0.014–0.037), but are consistent with the lack of association in a NZ Māori tribe (Ngāti Rakaipaaka, n = 110, *P* > 0.8) that was also reported by Myles et al. [4] as well as a small Tongan population reported by Kimura et al. [14]. The results from these multiple studies when considered together provide no repeated evidence for a functional role of this locus for traits such as T2D, BMI or gout in extant Māori or Samoan populations. However, it is possible that non-additive interaction with unmeasured environmental exposures may obscure evidence for main effect association of Gly482Ser with metabolic phenotypes.

Based on the lack of evidence of association between the Gly482Ser variant with BMI and correlated diseases, as well as lack of evidence of association with any of the tests for selection, we conclude that this study does not support natural selection at either the Gly482Ser variant, or the *PPARGC1A* region in general. More extreme frequencies of the derived allele in the Polynesian populations can alternatively be explained by genetic drift associated with ancestral population bottlenecks during colonisation. Further, the lack of robust direct candidate gene-based evidence for the thrifty gene hypothesis in light of increasing evidence for selective influence of infectious disease-causing agents on genome composition [35] and other factors such as diet, responses to climate, and skin colour collectively increase doubt regarding ongoing validity of this hypothesis. This is supported by no global of evidence for selection at 65 T2D loci with nominal evidence for selection at individual loci driven by an equal measure of T2D protective and risk haplotypes [31].

## Additional files

Additional file 1: Table S1. Fourteen previously detected combinations of populations and genes from Voight et al. (2006) were analysed using the *selscan* software package (Szpiech and Hernandez 2014) as positive controls to provide insight into possible power of detection of signatures of selection. Seven of the previously detected 14 associations were repeated, where we defined evidence of selection as >1 SNP exceeding the 5% threshold of iHS values. For two genes (*LCT* in both CEU and GBR populations and *SLC44A5* in CHS and CHB populations) showed evidence of selection in populations with similar ancestry (Caucasian and Asian, respectively). Evidence of selection for one other gene (*SNTG1*) was observed in only one of two populations with the same ethnicity. (DOCX 25 kb)
Additional file 2: Figures S1a-c. Tajima’s *D* calculated across chromosome 4 using a 30 kbp sliding window by population. Chromosome 4:22.7–24.9 Mbp is shown with chromosome mean (blue) and 2.5%, 97.5% quantiles (purple) from Table 4 marked. Location of *PPARGC1A* is marked in red. Rs8192678 is marked by a red dashed line. **Figure S1b** Tajima’s *D* calculated across chromosome 4 using a 5 kbp sliding window by population. Chromosome 4:22.7–24.9 Mbp is shown with chromosome mean (purple) and 2.5%, 97.5% quantiles (purple) from Table 4 marked. Location of *PPARGC1A* is marked in red. Rs8192678 is marked by a red dashed line. **Figure S1c** Tajima’s *D* calculated across chromosome 4 using a 1 kbp sliding window by population. Chromosome 4:22.7–24.9 Mbp is shown with chromosome mean (blue) and 2.5%, 97.5% quantiles (purple) from Table 4 marked. Location of *PPARGC1A* is marked in red. Rs8192678 is marked by a red dashed line. (ZIP 1473 kb)
Additional file 3: Figures S2a-c. Fay and Wu’s *H* calculated across chromosome 4 using a 30 kbp sliding window. Chromosome 4:22.7–24.9 Mbp is shown with chromosome mean (blue) and 2.5%, 97.5% quantiles (purple) from Table 4 marked. Location of *PPARGC1A* is marked in red. Rs8192678 is marked by a red dashed line. **Figure S2b** Fay and Wu’s *H* calculated across chromosome 4 using a 5 kbp sliding window. Chromosome 4:22.7–24.9 Mbp is shown with chromosome mean (blue) and 2.5%, 97.5% quantiles (purple) from Table 4 marked. Location of *PPARGC1A* is marked in red. Rs8192678 is marked by a red dashed line. **Figure S2c** Fay and Wu’s *H* calculated across chromosome 4 using a 1 kbp sliding window. Chromosome 4:22.7–24.9 Mbp is shown with chromosome mean (blue) and 2.5%, 97.5% quantiles (purple) from Table 4 marked. Location of *PPARGC1A* is marked in red. Rs8192678 is marked by a red dashed line. (ZIP 841 kb)

## Abbreviations

BMI: Body mass index
CEU: Utah residents with Northern and Western European ancestry
CHB: Chinese in Beijing, China
CHS: Southern Han Chinese, China
CI: Cook Island
GBR: British in England and Scotland
iHH: Integrated haplotype homozygosity
iHS: Integrated haplotype homozygosity score
NZ: New Zealand
SNP: Single nucleotide polymorphism
T2D: Type 2 diabetes
WGS: Whole genome (re)sequence
XP-EHH: Cross population extended haplotype homozygosity
YRI: Yoruba in Ibadan, Nigeria
