# Supplementary material for: Lack of direct evidence for natural selection at the candidate thrifty gene locus, PPARGC1A
Source: BMC Med Genet. 2016 Nov 15;17:80. doi: 10.1186/s12881-016-0341-z (PMC5111290; doi:10.1186/s12881-016-0341-z)
Supplement: Additional file 1: Table S1. — Fourteen previously detected combinations of populations and genes from Voight et al. (2006) were analysed using the selscan software package (Szpiech and Hernandez 2014) as positive controls to provide insight into possible power of detection of signatures of selection. Seven of the previously detected 14 associations were repeated, where we defined evidence of selection as >1 SNP exceeding the 5% threshold of iHS values. For two genes (LCT in both CEU and GBR populations and SLC44A5 in CHS and CHB populations) showed evidence of selection in populations with similar ancestry (Caucasian and Asian, respectively). Evidence of selection for one other gene (SNTG1) was observed in only one of two populations with the same ethnicity. (DOCX 25 kb) [file 12881_2016_341_MOESM1_ESM.docx]

Supplemental Material

# Lack of Direct Evidence for Natural Selection at the Candidate Thrifty Gene Locus, *PPARGC1A*

#

Murray Cadzow^1,2^, Tony R Merriman^1,2^, James Boocock^1,2^, Nicola Dalbeth^4^, Lisa Stamp^5^, Michael A Black^1,2^, Peter Visscher^2,6,7^, Phillip L Wilcox^1,2,3,8**^

^1^ Department of Biochemistry, University of Otago, Dunedin, New Zealand

^2^ Virtual Institute of Statistical Genetics, New Zealand

^3^ Scion (New Zealand Forest Research Institute Ltd), 49 Sala Street, Rotorua, New Zealand

^4^ Department of Medicine, University of Auckland, Auckland, New Zealand

^5^ Department of Medicine, University of Otago, Christchurch, New Zealand

^6^ The Queensland Brain Institute, University of Queensland, Brisbane, Australia

^7^ University of Queensland Diamantina Institute, University of Queensland, Translational Research Institute (TRI), Brisbane, Australia

^8^ Department of Mathematics and Statistics, University of Otago, Dunedin, New Zealand

### Selection analyses

Variant data for the OMNIexpress and AXIOM chips were filtered for minor allele frequency (> 0.01), Hardy-Weinberg equilibrium (*P* > 0.001), and non-missingness (> 0.99) using PLINK. Shapeit2 v0.778 was used to phase haplotypes and the most likely haplotype was selected for each sample. Phased haplotypes were imputed using the Sanger Imputation server (<https://imputation.sanger.ac.uk>) utilising the 1000 Genomes Project Phase 3 reference panel. Imputed genotypes were then filtered to remove an impute information score less than 0.8. Remaining markers present in all data sets were then used for the selection pipeline after excluding non-biallelic sites and INDELs. The imputed genotypes for Maori, Samoan, and the 1000 Genomes populations had INDELs removed.

Prior to imputation, (a) chromosome 4 AXIOM (i.e., Māori) had 32185 SNPs with mean(sd) between SNPs of 5929 (22384) bp, compared to post-imputation there were 313474 SNPs with mean 609 (5758) bp, and (b) chromosome 4 OMNI (i.e., Samoan) had 28149 SNPs with mean(sd) between SNPs of 6780 (23776) bp, compared to imputation there were 323547 SNPs with mean(sd) between SNPs 590 (5458) bp.

The population data were then used for:

a) haplotype recoding as ancestral or derived based on ancestral state from the 6-way primate Enredo-Pecan-Ortheus alignment FASTA from Ensembl (Flicek et al., 2012, ftp://ftp.ensembl.org/pub/release-66/fasta/ancestral_alleles/homo_sapiens_ancestor_GRCh37_e66.tar.bz) which was then used to calculate iHS using a genetic map. Integrated haplotype homozygosity scores were calculated and normalised using *selscan*. Gap penalties were introduced with a penalty of 20 for every 20 kbp of gap and a calculation cut-off at 200 kbp for larger gaps as implemented by Voight *et. al.* (2006);

b) calculating chromosome-wide Tajima's *D* in non-overlapping sliding windows of 1 kbp, 5 kbp, and 30 kbp using VCFtools; and

c) calculating Fay and Wu's *H* using the ancestral state as the outgroup in Variscan, with the same window sizes as used to calculate Tajima's *D*. Populations were combined into a single VCF and F_ST_ was calculated pair-wise by population in non-overlapping sliding windows of 1 bp and 5 Mbp across the chromosome using the Weir and Cockerham method as implemented by VCFtools. XP-EHH was calculated and normalised pair-wise by population using selscan. A threshold of > 3.29 (top 1% of SNPs) for |iHS| or |XP-EHH| was used to establish significance. The mean, 2.5% and 97.5% quantiles were calculated for the chromosome for Tajima's *D*, Fay and Wu's *H* and F_ST_, with the quantiles used to assess significance.

**Supplemental Table 1.** Fourteen previously detected combinations of populations and genes from Voight et. Al. (2006) were analysed using the *selscan* software package (Szpiech and Hernandez 2014) as positive controls to provide insight into possible power of detection of signatures of selection. Seven of the previously detected 14 associations were repeated, where we defined evidence of selection as >1 SNP exceeding the 5% threshold of iHS values. For two genes (*LCT* in both CEU and GBR populations and *SLC44A5* in CHS and CHB populations) showed evidence of selection in populations with similar ancestry (Caucasian and Asian, respectively). Evidence of selection for one other gene (*SNTG1*) was observed in only one of two populations with the same ethnicity.

| Gene | Population | Total SNPs above 5% Threshold | Total SNPs in region | Total SNPs above 5% Threshold | Total SNPs in region3 |
| --- | --- | --- | --- | --- | --- |
|  |  | **Gene Unpadded*** | | **Gene Padded +/-100kb** | |
| *SLC44A5* | CHB | 12 | 460 | 12 | 490 |
| *SLC44A5* | CHS | 9 | 359 | 9 | 389 |
| *LCT* | CEU | 11 | 60 | 52 | 270 |
| *LCT* | GBR | 18 | 59 | 60 | 276 |
| *SNTG1* | CEU | 0 | 1143 | 0 | 1434 |
| *SNTG1* | CHB | 0 | 1122 | 0 | 1526 |
| *SNTG1* | CHS | 18 | 1130 | 29 | 1534 |
| *SNTG1* | GBR | 3 | 1374 | 3 | 1665 |
| *SNTG1* | YRI | 0 | 1863 | 0 | 2459 |
| *SPAG4* | CEU | 0 | 2 | 1 | 298 |
| *SPAG4* | GBR | 0 | 2 | 0 | 243 |
| *SPAG4* | YRI | 0 | 4 | 0 | 356 |
| *SYT1* | YRI | 15 | 646 | 15 | 808 |
| *NCOA1* | YRI | 0 | 165 | 0 | 472 |

*refers to the protein coding and intronic regions of the gene only.

### FIGURES

**Supplemental Figure 1a.** Tajima's *D* calculated across chromosome 4 using a 30 kbp sliding window by population. Chromosome 4:22.7-24.9 Mbp is shown with chromosome mean (blue) and 2.5%, 97.5% quantiles (purple) from Table 4 marked. Location of *PPARGC1A* is marked in red. Rs8192678 is marked by a red dashed line.

**Supplemental Figure 1b** Tajima's *D* calculated across chromosome 4 using a 5 kbp sliding window by population. Chromosome 4:22.7-24.9 Mbp is shown with chromosome mean (purple) and 2.5%, 97.5% quantiles (purple) from Table 4 marked. Location of *PPARGC1A* is marked in red. Rs8192678 is marked by a red dashed line.

**Supplemental Figure 1c** Tajima's *D* calculated across chromosome 4 using a 1 kbp sliding window by population. Chromosome 4:22.7-24.9 Mbp is shown with chromosome mean (blue) and 2.5%, 97.5% quantiles (purple) from Table 4 marked. Location of *PPARGC1A* is marked in red. Rs8192678 is marked by a red dashed line.

**Supplemental Figure 2a** Fay and Wu's *H* calculated across chromosome 4 using a 30 kbp sliding window. Chromosome 4:22.7-24.9 Mbp is shown with chromosome mean (blue) and 2.5%, 97.5% quantiles (purple) from Table 4 marked. Location of *PPARGC1A* is marked in red. Rs8192678 is marked by a red dashed line.

**Supplemental Figure 2b** Fay and Wu's *H* calculated across chromosome 4 using a 5 kbp sliding window. Chromosome 4:22.7-24.9 Mbp is shown with chromosome mean (blue) and 2.5%, 97.5% quantiles (purple) from Table 4 marked. Location of *PPARGC1A* is marked in red. Rs8192678 is marked by a red dashed line.

**Supplemental Figure 2c** Fay and Wu's *H* calculated across chromosome 4 using a 1 kbp sliding window. Chromosome 4:22.7-24.9 Mbp is shown with chromosome mean (blue) and 2.5%, 97.5% quantiles (purple) from Table 4 marked. Location of *PPARGC1A* is marked in red. Rs8192678 is marked by a red dashed line.
